# An evaluation of multi-species empirical tree mortality algorithms for dynamic vegetation modelling

**DOI:** 10.1038/s41598-021-98880-2

**Published:** 2021-10-06

**Authors:** Timothy Thrippleton, Lisa Hülsmann, Maxime Cailleret, Harald Bugmann

**Affiliations:** 1 Department of Environmental Systems Science, Forest Ecology, Swiss Federal Institute of Technology (ETH Zurich), Universitätstrasse 16, 8092 Zürich, Switzerland; 2 Forest Resources and Management, Sustainable Forestry, Swiss Federal Research Institute (WSL), Zürcherstrasse 111, 8903 Birmensdorf, Switzerland; 3 Theoretical Ecology Lab, Faculty of Biology and Pre-Clinical Medicine, University of Regensburg, Universitätsstraße 31, 93053 Regensburg, Germany; 4 INRAE, Aix-Marseille University, UMR RECOVER, 3275 Route de Cézanne, CS 40061, Aix-en-Provence Cedex 5, France

**Keywords:** Ecology, Ecological modelling

## Abstract

Tree mortality is key for projecting forest dynamics, but difficult to portray in dynamic vegetation models (DVMs). Empirical mortality algorithms (MAs) are often considered promising, but little is known about DVM robustness when employing MAs of various structures and origins for multiple species. We analysed empirical MAs for a suite of European tree species within a consistent DVM framework under present and future climates in two climatically different study areas in Switzerland and evaluated their performance using empirical data from old-growth forests across Europe. DVM projections under present climate showed substantial variations when using alternative empirical MAs for the same species. Under climate change, DVM projections showed partly contrasting mortality responses for the same species. These opposing patterns were associated with MA structures (i.e. explanatory variables) and occurred independent of species ecological characteristics. When comparing simulated forest structure with data from old-growth forests, we found frequent overestimations of basal area, which can lead to flawed projections of carbon sequestration and other ecosystem services. While using empirical MAs in DVMs may appear promising, our results emphasize the importance of selecting them cautiously. We therefore synthesize our insights into a guideline for the appropriate use of empirical MAs in DVM applications.

## Introduction

Tree mortality is a key driver of the dynamics and functioning of forest ecosystems worldwide, and it is becoming increasingly important with ongoing climate change^1–3^. Despite decades of intensive research, individual-level tree mortality unrelated to severe disturbances also referred to as ‘background mortality’^4^ remains insufficiently understood due to its multi-causal nature and high complexity at the physiological level^5,6^. This incomplete understanding is reflected in the uncertainty of mortality representations in all approaches to simulate forest dynamics using vegetation models (DVMs), from physiological, process-based^7^ to theoretical or phenomenological approaches^8^. Since DVMs are highly sensitive to the formulation of mortality algorithms (MAs)^9,10^, this uncertainty has far-reaching consequences for projections of long-term forest dynamics and ecosystem functions from local to global scales^10,11^. A better representation of mortality at both individual and species levels, a stronger connection to data, together with the suitability of the approaches for climate change applications are considered to be key for improving projections of forest development under global change^12–15^.

Over the last years, continuously growing datasets of tree-ring width and forest inventories were established, providing a wealth of information on tree demography. Hence, these datasets are considered as a promising foundation for developing empirically-based MAs for DVM applications^13,16,17^. Among the empirical MAs, inventory-based MAs provide representative mortality probabilities and offer a particularly broad portfolio for different tree species^18^. In spite of the growing number of empirical MAs, selecting an appropriate MA for inclusion in a DVM remains a highly challenging task^10^, since their behaviour depends on the underlying dataset as well as on their structure, i.e., the particular predictor variables considered^19,20^.

A common predictor used in almost all empirical MAs is tree size (typically expressed as diameter at breast height, DBH), since tree mortality rates are usually highest for small as well as for tall, old trees^19^. Some empirical MAs rely even solely on tree size (‘Size-only MAs’), as it can explain a large part of the variability in mortality between individual trees^20^. Inventory-based MAs can furthermore be categorized based on their representation of environmental effects and competition. While one group of MAs considers competition directly via an index (‘CI-based’ MAs), other MAs represent competition and environmental effects indirectly via altered growth rates (‘Growth-based’ MAs), assuming that tree growth is an integrative indicator of tree vitality^21^. As recently demonstrated in a single-species application by Thrippleton, et al.^22^, this difference in MA structure can be of key importance in DVM applications, and has the potential of causing ecologically implausible projections in a climate change context. Thus, despite the increasing availability of empirical MAs, there is a substantial risk that inadequate MAs are employed in DVMs, which could lead to flawed long-term projections of ecosystem dynamics and functioning (e.g., carbon sequestration^11^).

A particular aspect of concern in the context of climate change is the scarcity of knowledge about applications of MAs for multiple, ecologically different species in DVMs. Tree species can differ substantially in mortality rates and in their mortality responses to changing environmental conditions^12,23,24^. Multi-species analyses of tree mortality conducted outside a DVM framework have emphasized the key importance of species’ ecological characteristics (i.e., their functional traits), with a clear distinction between light-demanding pioneer species and more shade-tolerant late-successional species^25^. In general, early-successional pioneer species tend to be shorter lived and are more light-demanding than late-successional species^26^, which makes them generally more sensitive to competition than late-successional species^27^. The main reason for this pattern has been attributed to different adaptations to survive under shaded environments^28,29^, e.g., physiological adaptations and differences in carbon storage strategies^25,27^. In the face of climate change, tree species differ profoundly in their capacity to survive times of heat and low water supply^6,30,31^. Already under contemporary levels of climate change, drought-intolerant species show substantially higher levels of mortality^32,33^, a pattern that is likely to be exacerbated by future climate change^1,24^.

Previous studies applying empirical MAs within a DVM framework have either focused on evaluating dynamics without considering different types of empirical MAs (e.g., geographic origin, structure and parameterization) or without climate change^18,29,34^, compared MAs embedded in vastly different models of vegetation dynamics^10^, or focused on a single species only^22^. Therefore, a systematic analysis of a wide range of multi-species empirical MAs is needed to evaluate whether these MAs (1) capture basic patterns of mortality under present and future climate in accordance with the species’ ecological characteristics; and (2) produce consistent patterns of mortality within the same species. It is furthermore of key importance to evaluate the long-term performance of multi-species MAs in comparison to empirical data, since the effect of the mortality representation in DVMs typically increases with simulation time^35^ and differences in the performance of empirical MAs become evident particularly in century-scale applications^18^.

Here, we perform a systematic and comprehensive analysis of inventory-based MAs for a diverse set of species within a single, consistent DVM framework. Using the forest gap model ForClim^35,36^, we explored MA behaviour for six common European tree species (*Pinus sylvestris L., Picea abies, Abies alba, Betula pendula, Quercus petraea, Fagus sylvatica*) that feature widely different ecological characteristics, ranging from light-demanding to shade-tolerant coniferous and broadleaved species. For these species, we included a total of 22 empirical MAs based Hülsmann, et al.^37^, who reviewed individual-based mortality models derived from inventory data in the absence of exogenous disturbances. The original MA of the DVM ForClim (a growth-based, theoretical MA^9^) was additionally included as a baseline for comparisons with empirical MAs, which are expected to be more accurate than the original MA.

Following the approach of Thrippleton, et al.^22^, we analysed MA behaviour (i.e., simulated mortality response to changing competition and climate conditions) (1) under present climate at a typical mesic and a more xeric site in Switzerland, and (2) under three climate change scenarios (i.e., a ‘warmer’, ‘warmer moister’ and ‘warmer drier’ climate), and (3) we evaluated DVM projections by comparing the stand structure emerging from simulations of a dynamic equilibrium under present climate with empirical data from a range of old-growth forests across Europe. Our baseline expectation was to find consistent mortality patterns simulated by the different MAs of all structural groups (CI-based, Growth-based, Size-only^37^), mortality responses to climate change in accordance with species’ ecological characteristics^4^, and simulated forest structures within ranges observed for old-growth forests^35^. As a final step, we synthesize our findings together with insights gained from previous studies into a guideline for the choice of MAs in various DVM applications.

## Results

### MA behaviour under present climate

**Figure 1:**
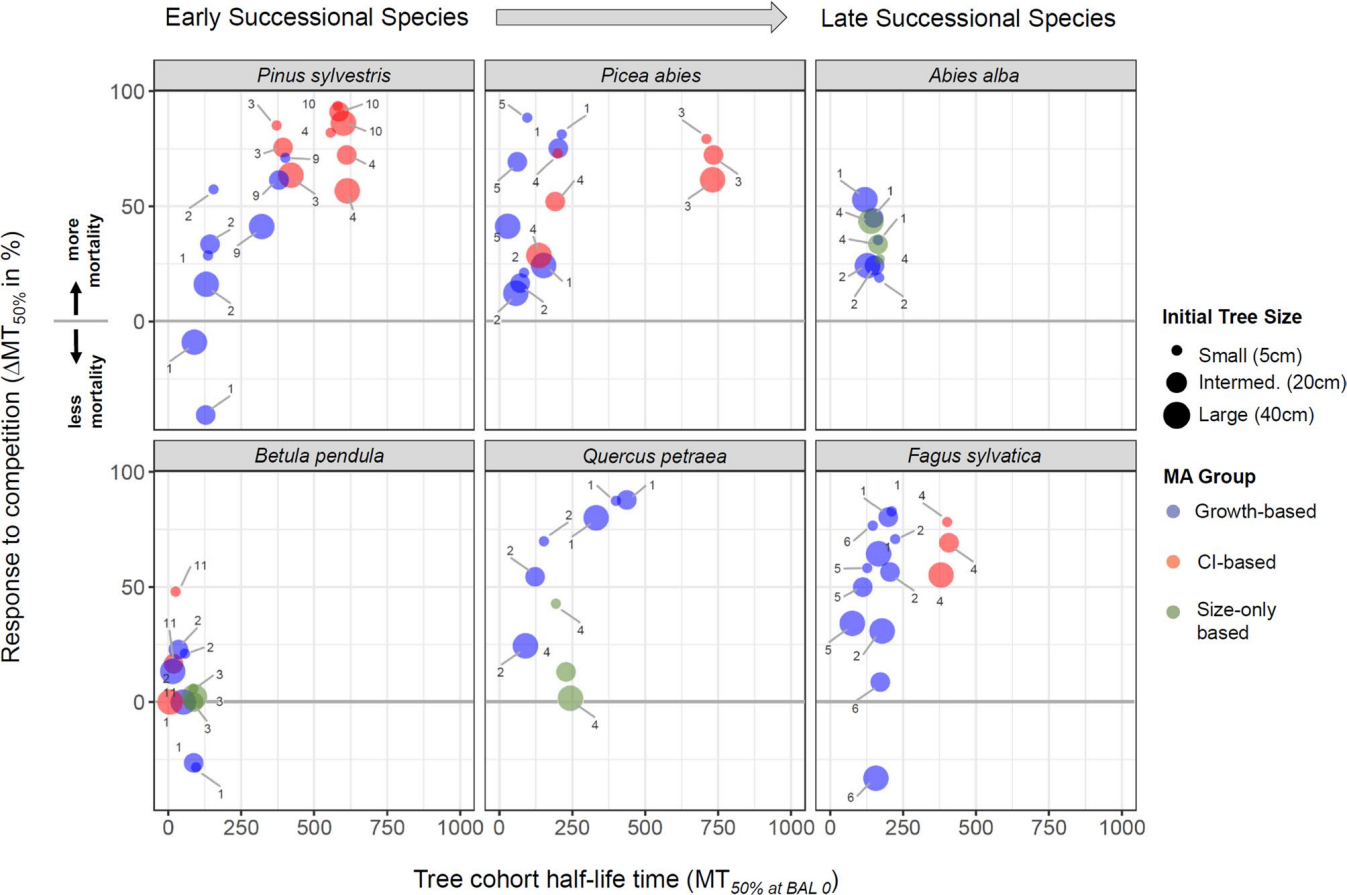
Comparison of model behaviour based on the projected tree cohort half-life times (MT_50%_, time (years) until 50% of the initially present trees died) and the response to competition (∆MT_50%_, i.e. the relative change of MT_50%_ in percentage in response to a increase in BAL by 10 m^2^ ha^−1^ compared to a ‘no competition’ scenario) for six tree species at the mesic site (Bern). Note that for response to competition, positive values indicate a relative increase in mortality and negative values a decrease in mortality. Symbol size indicates the three initial tree sizes used in the simulations, and symbol color indicates MA structure (differentiating between ‘Growth-based’, ‘Competition-index’ (CI)-based and ‘Size-only’ based MAs). Numbers refer the MA sources: 1: ForClim default MA (based on theoretical assumptions^9^), 2: Hülsmann, et al.^18^ , 3:Eid and Tuhus^38^, 4: Monserud and Sterba^39^ , 5: Dursky^40^ , 6: Holzwarth, et al.^20^ , 7: Trasobares, et al.^41^ , 8: Crecente-Campo, et al.^42^ , 9: Palahi, et al.^43^ , 10: Bravo-Oviedo, et al.^44^, 11: Fridman and Ståhl^45^ . Results for the more xeric study site (Basel) were very similar (see Appendix S2.1).

Across the six species, large differences in simulated mortality patterns occurred under present climatic conditions (Fig. 1). MA structure (i.e., the explanatory variables considered to predict mortality) was an important determinant of the simulated tree cohort half-life time (expressed as MT_50%_, i.e., the mean time until 50% of the initially present trees died) and response to competition (expressed as ∆MT_50%_, the relative percentage change in MT_50%_ compared to a ‘no competition’ scenario). With the exception of *Betula pendula*, ‘CI-based MAs showed higher MT_50%_ than ‘Growth-based’ and ‘Size-only’-based MAs, and were highly sensitive to changes in competition (Fig. 1). This pattern occurred consistently at both sites (i.e., the moister site Bern, Fig. 1, and the more xeric site Basel, Fig. S2.1) and for all investigated tree sizes (Fig. 1). The difference between ‘CI-based’, ‘Growth-based’ and ‘Size-only-based’ MAs was most pronounced for low values of competition and decreased with increasing competition intensity (Fig. S2.4). A notable exception to the difference between the MA groups was found for *Picea abies*, where the ‘CI-based’ MA by Monserud & Sterba (1999) behaved similarly as the ‘Growth-based’ MAs (Fig. 1). At the species level, no clear distinction in terms of tree cohort half-life times and response to competition was evident between early- and late-successional species due to high within-species variability (Fig. 1 and Fig. S2.1).

### MA behaviour under climate change

**Figure 2:**
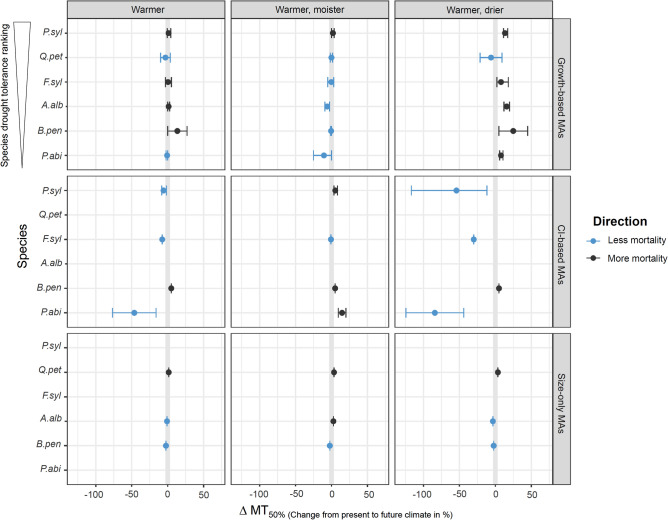
Mean change of MT_50%_ (∆MT_50%_ in percentage) under future climate relative to present climate conditions (for BAL of 10 m^2^/ha and ‘Medium size’ of initial trees) for different tree species (in order of descending drought tolerance, based on Huber, et al.^35^) at the site ‘Basel’. Note that for ∆MT_50%_, positive values indicate more mortality and negative values less mortality compared to the present climate conditions. Bars indicate ranges between min and max values of different MAs, no bars indicate that only one MA was present in the respective group. Mortality responses were similar for different initial tree sizes and competition conditions, see Appendix S2.3–S2.8.

Simulations carried out for the three climate change scenarios at both study sites (mesic site ‘Bern’ and more xeric site ‘Basel’) showed relatively small changes in MT_50%_ for the ‘warmer’ and ‘warmer and moister’ scenario (e.g., mean ∆MT_50%_ of −3.4% and + 1%, respectively for Basel) compared to present climate conditions (Fig. 2, Fig. S2.2). At the more xeric site (Basel), a ‘warmer and drier’ climate however resulted in a considerable increase in mortality for the ‘Growth-based MAs’ (mean ∆MT_50%_ of + 11% over all ‘Growth-based’ MAs), while the opposite response occurred for the ‘CI-based’ MAs (less mortality, mean ∆MT_50%_ of −41%) (Fig. 2). Results for the mesic site (Bern) showed similar patterns, but of a much smaller magnitude (mean ∆MT_50%_ of + 1% and −7%, respectively, see Fig. S2.2). Not surprisingly, MAs based solely on tree size (‘Size-only’) showed only a minimal and inconsistent response to the CC scenarios (Fig. 2). At the level of the tree species, no clear relationship emerged between ∆MT_50%_ and species drought tolerance, even within the group of ‘Growth-based’ MAs (Fig. 2).

### MA performance: empirical evaluation

**Figure 3:**
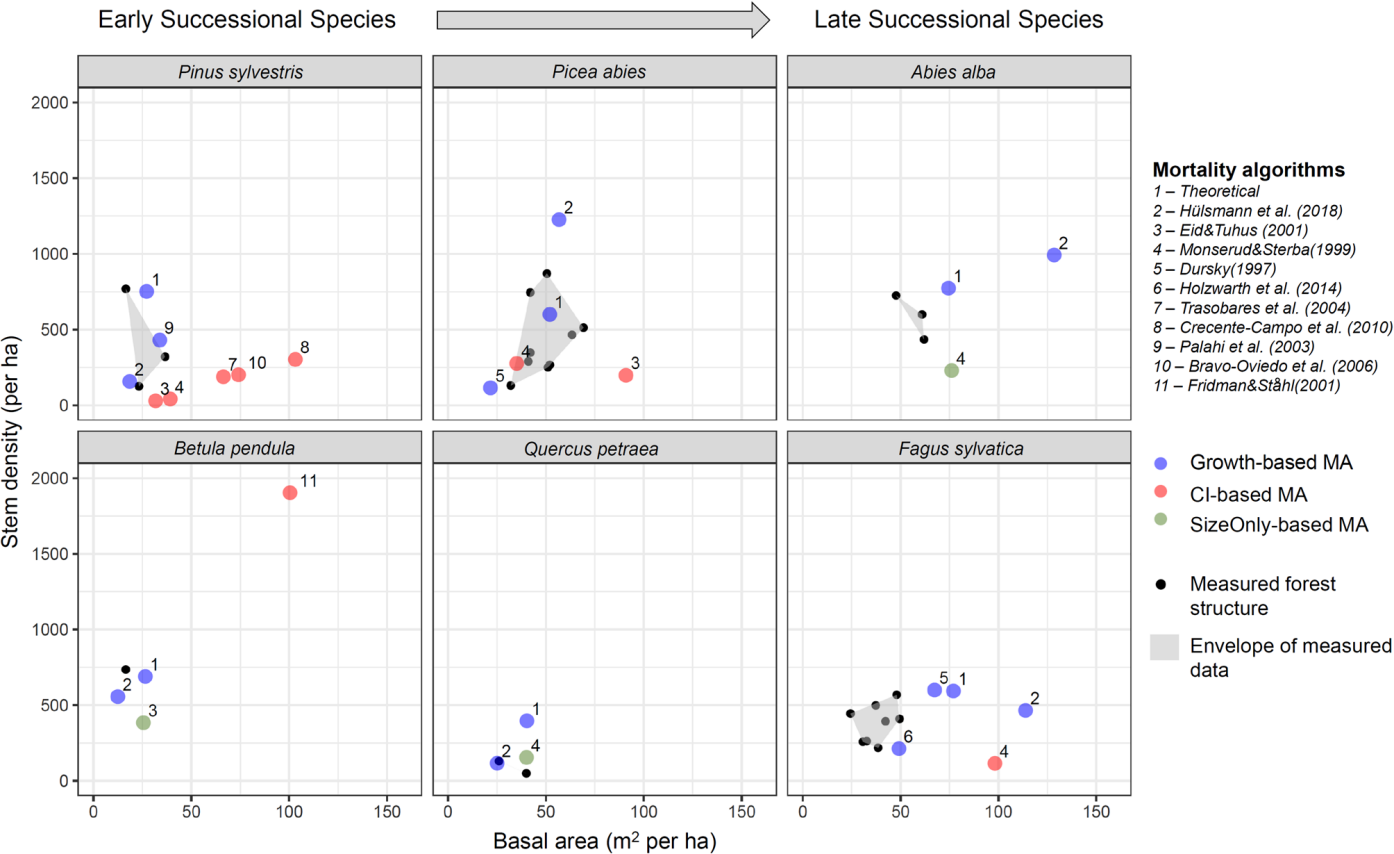
Comparison of forest structure (basal area and stem density, dbh > 7 cm) simulated by ForClim in dynamic equilibrium at the European study sites (see Appendix S1 and Fig. S1.1 for details) with measured data from old-growth forests (black dots indicating empirical measurements and grey areas indicating the envelope of measured data). Sources for empirical data for the different species are provided in Appendix S1.

Simulated stand structure (basal area and stem density) in dynamic equilibrium showed large differences between MAs and partly implausible stand structures when compared with empirical data from old-growth forests (Fig. 3). Substantial overestimations to the point of implausible basal areas for old-growth forest (e.g., > 100 m^2^ per ha) occurred for MAs of the ‘CI-based’ as well as for the ‘Growth-based’ group. When comparing the percentage bias between simulated and observed forest structure across all species, ‘Growth-based’ MAs showed an overprediction of mean basal area by 36% and ‘CI-based’ MAs an overprediction of 162% across all species (Table S2.9). At the level of individual species, implausible overestimations of basal area were found for all species, except for *Quercus petraea* (where however only 3 MAs were available).

## Discussion

In contrast to our expectations, the results showed (1) high variability and inconsistent mortality patterns between alternative MAs for multiple species under current climatic conditions; (2) opposite responses to climate change whose directions were associated with MA structure but independent of species characteristics; and (3) high variability of simulated forest structure for all species, which in most cases deviated substantially from empirically supported ranges. We therefore recommend that empirical MAs for multiple species should be applied in DVMs only if their behaviour has been tested for different scenarios in advance, particularly once they are applied in a climate change context. Below, we discuss each aspect in more detail and conclude with specific recommendations for selecting appropriate empirical MAs for DVM applications (Table 1).

Under present climatic conditions, several studies^12,37,46^ have shown that empirical MAs based on large inventory datasets are usually well-suited to capture the relationship of mortality with tree size, competition and site conditions for a wide range of species, thus making them promising candidates for improving the empirical foundation of DVMs^46^. A previous study by Hülsmann, et al.^37^ analysed empirical MAs from different sources outside a DVM framework and found consistent species-specific patterns of mortality despite large differences in the underlying methodology (i.e., model structure, parameterization), datasets and geographic origin. However, when applying MAs from the same sources as Hülsmann, et al.^37^ within a DVM framework, we found highly variable and largely inconsistent results at the level of individual species, indicating complex feedbacks between MAs and DVMs. Two major reasons complicate the straightforward application of empirical MAs in DVMs.

First and most evidently, the difference in MA structure (‘CI-based’, ‘Growth-based’, ‘Size-only based’) led to considerably different mortality responses in the DVM. CI-based MAs are typically well-suited to capture mortality primarily in response to the effects of changes in competition^19,44^, a major driver of mortality in dense young stands undergoing self-thinning, but less so in old-growth forests^47^. In contrast, ‘Growth-based’ MAs consider competition and other environmental factors via decreased changes in radial growth rates^46^, reflecting the fact that reduced growth is often linked to a higher mortality probability^16,48^. ‘Growth-based’ MAs thus appear to be more flexible in capturing mortality processes, e.g. in older life stages (for instance drought impacts in larger trees^31^) and thus may be structurally better suited for DVM applications over longer simulation intervals^22^.

A second reason for the large differences of MA performance lies in the datasets used for their calibration. In particular, some of the MAs in our study covered different diameter ranges^37^, which can lead to substantially different mortality predictions (e.g., due to different mortality causes for different life stages^20^). For instance, the calibration dataset for the MA of *Picea abies* by Monserud and Sterba^39^ included observations from large trees that featured a high mortality probability (i.e., a U-shaped function of DBH^12^), thus resulting in a more realistic basal area in the dynamic equilibrium simulations (Fig. 3). Similarly, the ‘Size-only’ based MAs included in our study were based on nation-wide datasets including also large trees (e.g., MAs for *Abies* and *Quercus*^39^), thus explaining their relatively good performance (i.e., basal area close to empirical observations) for the dynamic-equilibrium simulations.

In summary, we found that large differences in the datasets as well as in the structure and parameterization of the MAs overruled species-specific mortality responses in the DVM application under current climate. Although the MAs show species-specific patterns when applied within the range of their calibration data (e.g., the observed diameter range and stand structure) outside a DVM^37^, our findings emphasize that substantial extrapolation effects^23^ can occur when applying empirical MAs in a DVM framework^9,49^.

Under future warmer and drier climatic conditions, our expectation was to find mortality responses in accordance with the species’ ecological characteristics, in particular their drought tolerance^4,31^. In contrast, the observed response was driven mainly by MA structure. For ‘Growth-based’ MAs, the drought-induced reduction in growth under the ‘drier’ scenario resulted in an increase in mortality, which is in agreement with observations from irrigation experiments^50^, long-term monitoring^51^ and studies along environmental gradients^1,4,51^. For the ‘CI-based’ MAs, however, a growth reduction led predominantly to a decrease in competition intensity (relative to the present climate), which resulted in a decrease in mortality, since other environmental variables (e.g., site index) were insufficient to capture the effect of increasing drought^8^. This pattern has previously been found for *Pinus sylvestris*^22^, but its generality in the context of climate change responses has not been demonstrated in a DVM context for a wider suite of species.

The absence of a relationship between a tree species’ drought tolerance and its mortality response to climate change for the ‘Growth-based’ MA group is contrary to empirical evidence, which suggests drastically different mortality rates of different tree species under a warmer and drier climate^1^, such as a high drought-sensitivity of *Picea abies*^52^ or a lower sensitivity of the drought-tolerant species *Quercus petraea* and *Pinus sylvestris*^53^. The absence of this pattern in our analysis is likely related to the large differences in the datasets underlying the calibration of the MAs^37^, which can contain complex site-specific patterns^32^ or effects of biotic mortality agents which can confound the patterns related to single traits^6^. This problem can partly be overcome if MAs were derived using longer-term datasets that span larger regions, thus better capturing climate-change related signals such as drought^24,31^, and by using inverse calibration approaches to better integrate empirical data in a DVM framework^14^. Our findings thus underline the need for further research in developing empirical mortality models for multiple species with a suitable foundation for climate change applications in DVMs. Besides developing empirical MAs, the improvement of mechanistic, eco-physiological MAs that are applicable across species and regions will also be central, particularly for DVM applications under ‘no-analogue’ climatic conditions (e.g., unprecedented warming or drought conditions)^5,23^.

The evaluation of simulated forest dynamics with empirical data from old-growth forests revealed large variations in simulated forest structure, which in most cases substantially exceeded the observed data ranges. The partly large overestimation of basal area is of particular concern for DVM applications, since it implies similarly overestimated timber stocks in mature forests, an increasingly important topic in the context of the bio-economy^54^, and implausible values of carbon sequestration in living biomass^11^. Overestimation of basal area and biomass is furthermore problematic because DVMs are progressively used as tools for projecting biodiversity and ecosystem service provisioning of forests^55,56^ via indicators derived from simulated forest structure^57^. Underestimations of mortality for mature forests, as observed for some MAs in our study, may therefore lead to flawed conclusions for biodiversity (e.g., amount of deadwood^58^) and ecosystem service provisioning. Our findings thus highlight the importance of research on tree mortality in mature and old-growth stages, where besides competition, direct ecophysiological impacts that can be mediated strongly by biotic agents^6^ play a central role^47,59,60^.

Although inventory-based variables such as tree size and stand density can be more robust predictors of mortality rates than physiological indicators^15^, our study implies that the use of empirical MAs for multiple species based on large-scale datasets is no guarantee by itself for improving the robustness of DVM projections. We thus advise caution towards their use and emphasize that empirical MAs should be selected carefully according to the specific DVM application (cf. Table 1). Based on our findings, we consider three aspects to be critical for selecting an appropriate MA: (1) the timespan of the simulation (since the importance of the MA typically increases with DVM simulation time^61^), (2) the climatic conditions the DVM is applied to, and the degree to which they differ from the conditions the MA was developed for^22^, and (3) the species set used in the DVM simulation (see Table 1). We furthermore emphasize the importance of the spatial scale of the DVM application (e.g., from local to global) and region of application, which need to be considered when selecting empirical MAs (see also Adams, et al.^23^ and Hülsmann, et al.^37^). These topics were however beyond the scope of the present study and require further research. Given that the availability of empirical MAs is still limited, further MAs should be developed, preferably covering additional species while providing structurally similar models. Such developments of empirical MAs should aim at more flexible mortality models based on extensive, long-term datasets along broad environmental gradients^62^ that may become increasingly available via novel forest monitoring and data homogenization activities.

**Table 1:** Guideline for the use of empirical MAs in different DVM applications.

**1.) Which time scale is simulated?**	**Recommendation**
(a) Short-term (few decades)	Differences in MA types may be less important for short-term applications in mature forests. For young forests, the adequate representation of self-thinning by the MA should be tested beforehand, see Thrippleton et al.^22^
(b) Long-term (decades to centuries)	Preferentially use Growth-based MAs (sensitive to variations in environmental conditions). If applied to unmanaged conditions, use MAs developed from datasets including mature (and ideally old-growth) forests
**2.) Which climate is simulated?**	
(a) Present (historic) climate	Both Growth-based and CI-based MAs may be suitable if they were developed from datasets of a similar geographic region^37^ and based on large diameter ranges
(b) Climate change	Avoid CI-based and Size-only based MAs that miss a suitable climate-sensitive predictor, which can lead to unreasonable DVM behavior under intensified climatic stress
**3.) Which species are simulated?**	
(a) Only dominant (common) species	For species-specific MAs, structure and quality of the calibration dataset is crucial. Preferentially use Growth-based MAs developed from large-scale datasets (see 1b)
(b) Including rare species	Problems with data scarcity may be particularly high in DVM applications to species-rich forests or forests dominated by economically unimportant species. In the absence of suitable empirical MAs, an MA for a similar species or for the same species-group^62^ may be feasible. Alternatively (or generally in case of doubt), a MA based on ecologically sound theoretical reasoning^9^ may provide a good option

We conclude that even small differences in species-specific mortality probabilities can lead to drastically different DVM projections, which can result in biased and erroneous projections of future forest structure^9^, successional trajectories, species composition^34^ and ecosystem functioning, e.g., carbon uptake^11^ . A prudent selection and further development of empirical MAs is therefore an important step towards improving the robustness of the projections of DVMs, from the stand to the global scale. Considering the recent rapid increase in global datasets about tree demography^24,63^, empirical MAs have a large potential in bridging the knowledge gaps in mechanistic understanding and considerably improve future DVM projections alongside process-based physiological mortality models^23^.

## Methods

### Dynamic vegetation model ForClim

The study was conducted using the DVM ForClim, version 4.0.1^35^, which was originally developed for the simulation of forest dynamics in Central Europe and has been continuously extended for a wide range of applications (short- and long-term simulations of managed and unmanaged forests) across Europe and beyond^61,64^. ForClim is based on the so-called ‘gap model’ approach^65^ and shares large conceptual similarity with many other DVMs^66^. In this approach, growth, mortality and regeneration of tree cohorts (i.e., groups of trees of the same age and species) are simulated in response to environmental conditions within small patches, based on the theory of patch dynamics^67^. Tree growth is based on the concept of ‘constrained optimum growth’ ^68^, which means that growth under optimum conditions is reduced by environmental constraints (i.e., availability of light, water, temperature and nutrients) based on the species’ environmental tolerances (e.g., shade- or drought-tolerance). Tree mortality is by default expressed via a ‘theoretical’ MA, representing a combination of stress-related and size-dependent mortality^35^, see also Appendix S1.

### Empirical mortality algorithms (MAs)

We investigated a total of 22 inventory-based empirical MAs for six species (*Pinus sylvestris, Picea abies, Abies alba, Betula pendula, Quercus petraea, Fagus sylvatica*) from various sources^18,20,38–45^. All empirical MAs were based on logistic regression models^69^ where mortality probability (*p*) of a tree *i* is expressed as

1 $$p_{i} = {\text{ logit}}^{ - 1} (X_{i} b) = \exp (X_{i} b)/(1 + \exp (X_{i} b))$$

with *X*_*i*_ denoting the design matrix of the linear predictors and *β* the respective parameter vector. Almost all MAs use tree size (typically diameter at breast height, DBH) as a predictor variable and treat competition either directly using a competition index (typically basal area of larger trees, BAL; ‘CI-based’ MAs,) or indirectly using tree radial growth as a predictor (‘Growth-based’ MAs)^19^. Similarly, the effect of environmental conditions is included indirectly in the case of the Growth-based MAs and often directly via a site index in the case of the CI-based MAs. Site index is a typical measure of site quality in forestry and is expressed as the expected height of trees at a certain reference age^19^. An overview of all empirical MAs, their origin, diameter ranges and respective predictors is provided in Appendix S1, Table S1.1.

### Simulation setup

We systematically evaluated the behaviour of MAs in the DVM ForClim in three steps, following the protocol developed in Thrippleton, et al.^22^: (1) under present climate, (2) under climate change scenarios and (3) in simulations of dynamic equilibrium under the current climate, to be compared with empirical data from old-growth forests across Europe.

For steps (1) and (2), a full factorial design with the factors competition, tree size and climate was used in order to test the MAs for a wide range of conditions under clearly defined boundary conditions. For this purpose, a cohort of initial trees was planted at the beginning of the simulation (250 trees per ha, see Thrippleton, et al.^22^) and tracked over the course of 1000 years of simulation time under various conditions of tree size (with the levels ‘Small’: 5 cm; ‘Intermediate’: 20 cm and ‘Large’: 40 cm of DBH) and competition by larger trees (with the BAL levels: 0, 10, 20, 30, 40, 50 m^2^ per ha). For the simulation of competition by larger trees, taller trees (with a DBH of 20 cm for ‘Small’, 30 cm for ‘Intermediate’ and 50 cm for ‘Large’) were included as competitors in the simulation and initialized to produce a BAL of 10, 20, 30, 40, 50 m^2^ per ha. This setup was chosen to explore MA behaviour under a range of tree sizes and levels of competition in the calibration datasets of the MAs, see Appendix Table S1.1 and Hülsmann, et al.^37^ . To avoid confounding effects from tree regeneration, no regeneration was simulated in steps 1 and 2. Simulations under present climate and climate change were carried out at two sites, representing mesic conditions with near-optimal growing conditions (Bern) and more xeric conditions (Basel; see Appendix S1 for further details). At both sites, four climate scenarios were investigated (with the levels: ‘present’, ‘warmer’, ‘warmer, moister’ and ‘warmer drier’ climate), assuming a change of annual temperature and precipitation of + 4 °C for the ‘warmer’ scenario (no precipitation change), + 4 °C and + 20% precipitation for the ‘warmer, moister’ scenario as well as + 4 °C and −20% precipitation for the ‘warmer, drier’ climate scenario. For the transition to these future climates, a linear change of temperature and precipitation for 100 years (corresponding to the time period of year 2000 to 2100) was assumed until a new constant climate was reached for the rest of the simulation period (see Bircher, et al.^9^). The assumption of a temperature increase by 4 °C and a precipitation decrease by 20% was based on the ‘high impact’ RCP 8.5 projections until the end of the twenty-first century for Central Europe^70^. The assumption of a precipitation increase by 20% for the ‘warmer, moister’ scenario was based on the relatively large uncertainty regarding precipitation changes until the end of the twenty-first century, which also include the possibility of a precipitation increase at this order of magnitude^70^. For details about the present climate see Appendix Table S1.2, for details about the climate change simulations, cf. Appendix S1.

For step (3), the emergent forest structure in dynamic equilibrium was evaluated for each species, assuming monospecific stands. Simulations were started from bare ground (in contrast to the planted initial tree cohorts in steps 1 and 2) for a time span of 1000 years, under present climate, unmanaged conditions and assuming natural regeneration, as done in other long-term DVM applications^71^. Simulated forest structure at the end of the simulation time was compared to observed ranges (basal area and stem density) from largely undisturbed, old-growth forest reserves in Europe (see Appendix S1 for the respective references and Fig. S1.1 for their locations). For each species, the most representative study site was selected among the forest reserves available for each species to conduct the long-term simulations (see Table S1.2). The selection was based on site characteristics reflecting old-growth forest conditions and a clear dominance by the respective species of interest. Details about the study sites and the empirical data can be found in Appendix S1.

### Evaluation of MA behaviour and accuracy

MA behaviour for simulation steps 1 and 2 was characterized by two indicators as in Thrippleton, et al.^22^ : (1) the tree cohort half-life time (MT_50%_), defined as the mean time until 50% of the initially present trees died, and (2) the response of the MA to changing competition or climate conditions (∆MT_50%_), measured as the relative percentage change in MT_50%_ to a baseline scenario; positive ∆MT_50%_ values indicate higher mortality in comparison to a scenario without competition or climate change, respectively. For the evaluation of the long-term DVM simulations (step 3), simulated forest structure was compared to observed ranges in empirical measurements (basal area and stem density) from largely undisturbed, old-growth forest reserves (see above and Appendix S1 for further details). All analyses and visualizations were carried out using R version 3.6.1^72^.

## Supplementary Information

Supplementary Information.
