# Supplementary material for: An evaluation of multi-species empirical tree mortality algorithms for dynamic vegetation modelling
Source: Sci Rep. 2021 Oct 6;11:19845. doi: 10.1038/s41598-021-98880-2 (PMC8494886; doi:10.1038/s41598-021-98880-2)
Supplement: Supplementary file 1 — Supplementary Information. [file 41598_2021_98880_MOESM1_ESM.docx]

**Supplementary material S1 – Additional Material and Methods**

An evaluation of multi-species empirical tree mortality algorithms for dynamic vegetation modelling

Timothy Thrippleton^1,2*^, Lisa Hülsmann^3^, Maxime Cailleret^4^, Harald Bugmann^1^

1: Swiss Federal Institute of Technology (ETH Zurich), Department of Environmental

Systems Science, Forest Ecology, Universitätstrasse 16, 8092 Zürich, Switzerland.

2: Swiss Federal Research Institute (WSL), Forest Resources and Management, Sustainable

Forestry, Zürcherstrasse 111, 8903 Birmensdorf, Switzerland.

3: University of Regensburg, Faculty of Biology and Pre-Clinical Medicine,

Theoretical Ecology Lab, Universitätsstraße 31, 93053 Regensburg, Germany

4: INRAE, Aix-Marseille University, UMR RECOVER, 3275 route de Cézanne, CS 40061, Aix-en-Provence cedex 5, France

* Corresponding author (timothy.thrippleton@wsl.ch)

1. **Dynamic vegetation model ForClim**

As most other dynamic vegetation models (DVMs), ForClim explicitly considers demographic processes (i.e., establishment, growth and mortality) under the influence of biotic (e.g., competition) and abiotic (e.g., edaphoclimatic) conditions ^1,2^. ForClim belongs to the group of ‘forest gap models’, where vegetation dynamics is simulated on small patches of land (in the case of ForClim, 200 patches with a size of 800 m^2^), based on the theory of patch dynamics ^1,3^.

In ForClim, plant performance is driven by a number of environmental conditions: availability of light and water (soil moisture), nutrients, as well as temperature (annual degree day sum and winter temperature)^4^. Light extinction through the forest canopy is simulated dynamically based on stand structure and composition ^5^ via the Beer-Lambert law ^6^. Water availability is represented in the form of a drought-index, which is calculated via a water balance from climate data (monthly temperature and precipitation data) as well as soil water holding capacity ^7^. Furthermore, tree growth is limited by soil nutrient availability, i.e. nitrogen availability ^8^.

While larger-scale DVMs typically work on the level of plant functional types to cope with global plant diversity ^9-11^, ForClim focuses on dynamics at the species level ^5^. Each tree species is represented via a set of life-history traits ^12,13^. These include parameters related to growth (maximum growth rate, maximum height and diameter) as well as tolerances to climatic and other factors e.g., drought tolerance, minimum winter temperature tolerated, nitrogen and browsing tolerance, see ^13^. For a list of species-specific parameters, see Rasche, et al. ^14^ and Huber, et al. ^15^.

The model was originally developed for central European conditions ^5^, but has progressively been applied to other regions in Europe, as well as North America and Asia ^16-18^. The present study was conducted with ForClim V4.0.1, which features a revision of demographic processes (establishment, growth and mortality). This version has been thoroughly evaluated at various sites across Europe ^15^ using a pattern-oriented modeling approach ^19^. In the following, a brief description of the demographic processes used in ForClim version 4.0.1 is given, with a particular focus on the representation of mortality. Further details about this model version is provided in Huber ^13^ and Huber, et al. ^15^.

*Establishment*

Before tree establishment can occur, the suitability of environmental conditions for regeneration is checked in ForClim (e.g., in terms of light availability at the forest floor, maximum and minimum winter temperatures, degree-days, soil moisture and browsing pressure) ^5,20^. If conditions are suitable (i.e., are within the range of a species’ tolerances), saplings can establish at an average diameter of 1.26 cm at breast height. In ForClim V4.0.1, the revised establishment routine features a more realistic species-specific representation of tree regeneration. In contrast to previous model versions ^21^, environmental effects now also affect sapling numbers as well as the distribution of saplings among the species, depending on the species performance under the given environmental conditions. The new model version led to overall more realistic patterns of establishment when compared with empirical data of 16 sites across Europe ^15^. For the present study, establishment was only activated for the simulations in dynamic equilibrium (step 3), since the interaction between mortality and regeneration would have caused confounding effects in the study of MA behaviour in steps 1 and 2, see Thrippleton, et al. ^22^ for further explanations.

*Growth*

Growth in ForClim is based on the carbon budget approach by Moore ^23^ and takes into account plant carbon allocation strategies into height and diameter ^14^. The effect of multiple environmental constraints on tree growth is represented via the approach of ‘constrained optimum growth’ ^2^, where optimum annual growth is reduced by several growth-reducing factors (GFs), which consider environmental conditions and species-specific environmental tolerances ^5^. The growth-reducing factors for the multiple environmental constraints are combined into one annual growth-reduction factor (GRF), which determines actual growth in the respective year ^15^.

The ForClim version 4.0.1 used in the present study features a re-assessment of species-specific optimum growth-rate parameters (kG) as well as a revised calculation of GRF ^15^. The re-assessment of species-specific growth parameters was based on tree-ring chronologies of 15 European species from the International Tree-ring Data Bank (*https://www.ncdc.noaa.gov/data-access/paleoclimatology-data/datasets/tree-ring*). The revised calculation of GRF was evaluated at different study sites across Europe, covering various environmental conditions, and resulted in a general improvement of model performance ^13,15^.

*Mortality (‘theoretical MA’)*

The ‘theoretical MA’ of ForClim represents a combination of a size-dependent ‘background mortality’ component and a growth-dependent ‘stress-induced’ mortality component ^4,5,13^.

The representation of background mortality (*gP_Backgr_*), i.e., mortality caused by various unspecific mortality sources during a trees life (e.g., fungal or pathogen infections, insect infestations, mechanical damage, etc.) was revised for ForClim version 4.0.1 ^15^. While previous model versions assumed a constant background mortality based on a species maximum age ^4^, the ‘theoretical MA’ in the new model version employs a size-dependent background mortality, based on Manusch, et al. ^24^ and adapted by Huber, et al. ^15^. This approach features an increasing mortality probability for larger-sized trees, which is in accordance with various observations from old-growth forests ^25,26^.

The component of ‘background mortality’ is expressed as:

*gP_Backgr_* = 0.1 × (*DBH* / *kDMax*)^α^ (Eq. S1.1)

where the mortality probability (*gP_Backgr_*) depends on the diameter of a tree (*DBH*) relative to its species-specific maximum diameter value, *kDMax* ^12^ and α, a constant set to 2.3 for all species ^15^.

The representation of ‘stress induced’ mortality (*gP_Stress_*) in ForClim version 4.0.1 remained the same as in previous model versions. It is assumed that the mortality probability increases when the diameter increment drops below a threshold of either 10% of a species maximum increment or below an increment of 3 mm (representing ‘slow-growth’ conditions) for at least three consecutive years ^4^. Stress-related mortality is scaled so that a number of 10 consecutive years under these ‘slow-growth’ conditions results in a mortality probability of 99% ^4,5,27^.

Both mortality components are then combined into an overall mortality probability (*gP_Mort_*), which is calculated as:

*gP_Mort_* = *gP_Backgr._* + (1 - *gP_Backgr._*) × *gP_Stress_*  (Eq. S1.2)

Overall, the inclusion of a size-related background mortality resulted in more realistic simulations of stand structure than the previous age-related approach when compared to empirical data from forests at various sites across Europe ^15^.

*Implementation of empirical MAs*

For the present study, the ‘theoretical’ MA of ForClim was replaced by species-specific empirical mortality algorithms (MAs). All MAs were based on logistic regression models ^28,29^, expressing mortality probability (*p*) of tree *i* as:

*p_i_*  = logit^-1^ (*X_i_ β*) = exp (*X_i_ β*) / (1 + exp(*X_i_ β*)) (Eq. S1.3)

with *X_i_* denoting the design matrix of the linear predictor and *β* the parameter vector.

Based on the comprehensive compilation of Hülsmann, et al. ^30^, all those MAs for the species *Pinus sylvestris, Picea abies, Abies alba, Betula pendula, Quercus petraea, Fagus sylvatica* were included in the evaluation, which considered (1) individual-based mortality (i.e., mortality in the absence of large, exogenous disturbances, e.g. bark beetle infestations or windthrows), (2) were based on logistic regression models and (3) were derived from forest inventory datasets. Literature sources for the empirical MAs were: Dursky ^31^, Monserud and Sterba ^32^, Fridman and Ståhl ^33^, Palahi, et al. ^34^, Trasobares, et al. ^35^, Bravo-Oviedo, et al. ^36^, Crecente-Campo, et al. ^37^, Holzwarth, et al. ^26^, Hülsmann, et al. ^38^.

An overview of the 22 species-specific MAs is given in Table S1.1, including their country or region of origin, the range of tree sizes in the calibration datasets as well as predictor variables used in the MA. A detailed overview of the predictor variables and their parameter estimates is given in Hülsmann, et al. ^30^; further explanations about their implementation in ForClim is given in Thrippleton, et al. ^22^.

**Table S1.1** Empirical mortality algorithms (MAs) for the six investigated species, incl. their geographic origin (country or region of origin), size range in the calibration dataset (DBH in cm) as well as predictor variables. Abbreviations: CR: crown ratio, ELEV: Elevation (m a.s.l.), CON: Continentality (i.e., distance to sea in km), N: number of trees (per ha), PBA: percentage of basal area of the subject species, BA: stand basal area (m^2^ per ha). Further details about the MAs, their predictor variables and parameter estimates are given in Hülsmann, et al. ^30^.

| Species | Study | Country/Region | DBH_min_ | DBH_max_ | Predictor variables | | | | |
| --- | --- | --- | --- | --- | --- | --- | --- | --- | --- |
|  |  |  |  |  | Size | Competition-Index | Growth | Site-Index | Other |
| *Pinus sylvestris* | Eid and Tuhus (2001) ^39^ | Norway | 5 | 46 | x | x |  | x |  |
|  | Monserud and Sterba (1999) ^32^ | Austria | 5 |  | x | x |  |  | CR |
|  | Trasobares et al. (2004) ^35^ | Spain | 7.5 | 76 | x | x |  |  | ELEV,CON |
|  | Crecente-Campo et al. (2010) ^37^ | Spain | 5 | 49 | x | x |  |  | N |
|  | Palahi et al. (2003) ^34^ | Spain | 5 | 56 |  | x | x |  |  |
|  | Hülsmann et al. (2018) ^38^ | Germany, Switzerland | 4 | 78 | x |  | x |  |  |
|  | Bravo-Oviedo et al. (2006) ^36^ | Spain | 4.1 | 69 | x | x |  | x |  |
| *Picea abies* | Eid and Tuhus (2001) ^39^ | Norway | 5 |  | x | x |  | x | PBA |
|  | Monserud and Sterba (1999) ^32^ | Austria | 5 |  | x | x |  |  | CR |
|  | Dursky (1997) ^31^ | Germany |  |  | x |  | x | x |  |
|  | Hülsmann et al. (2018) ^38^ | Germany, Switzerland | 5 | 90 |  |  | x |  |  |
| *Abies alba* | Monserud and Sterba (1999) ^32^ | Austria | 5 |  | x |  |  |  | CR |
|  | Hülsmann et al. (2018) ^38^ | Germany, Switzerland | 4 | 90 | x |  | x |  |  |
| *Betula pendula* | Eid and Tuhus (2001) ^39^ | Norway | 5 |  | x |  |  |  |  |
|  | Fridman and Ståhl (2001) ^33^ | Sweden | 10 |  | x | x |  | x | BA, ELEV |
|  | Hülsmann et al. (2018) ^38^ | Germany, Switzerland | 4 | 80 | x |  | x |  |  |
| *Quercus petraea* | Monserud and Sterba (1999) ^32^ | Austria | 5 |  | x |  |  |  |  |
|  | Hülsmann et al. (2018) ^38^ | Germany, Switzerland | 4 | (>100) | x |  | x |  |  |
| *Fagus sylvatica* | Monserud and Sterba (1999) ^32^ | Austria | 5 |  | x | x |  |  | CR |
|  | Dursky (1997) ^31^ | Germany |  |  | x |  | x |  |  |
|  | Holzwarth et al. (2013) ^26^ | Germany | 1 | 110 | x |  | x |  |  |
|  | Hülsmann et al. (2018) ^38^ | Germany, Switzerland | 4 | 100 | x |  | x |  |  |

1. **Study sites and origin of empirical data**

The main analysis of MA behaviour (steps 1 and 2) was conducted at two study sites, representing optimum growing conditions (Bern) as well as a more xeric climatic conditions (Basel) to account for differential climate change impacts on sites with different drought-vulnerability ^40^. For the evaluation of the species-specific simulations in dynamic equilibrium (step 3), simulation results were compared to empirical data from old-growth forests at different sites across Europe.

Empirical data sources for old-growth forests were Brang and Heiri ^41^ for *Pinus sylvestris* at Pfynwald (Switzerland); Mason, et al. ^42^ for *Pinus sylvestris* at Glen More (Scotland); Rimle ^43^ for *Picea abies* at Scatlé, Deborence, Bödmerenwald and Seeliwald (Switzerland); Svoboda and Pouska ^44^ for *Picea abies* in the Bohemian forest (Czech Republic); Holeksa, et al. ^45^ for *Picea abies* in the Pol’ana reserve (Slovakia); Lamedica, et al. ^46^ for *Picea abies* in Suceava (Romania); Motta, et al. ^47^ for *Picea abies* in the Valbona forest reserve (Italy); Leibundgut ^48^ for *Abies alba* at Perucica and Pecka (Bosnia–Herzegovina); Korpel ^49^ for *Quercus petraea* in Boky (Slovakia); Bobiec ^50^ for *Quercus* stands in Białowieza (Poland); Petritan, et al. ^51^ for *Quercus* *petraea* and *Fagus sylvatia* at Runcu-Grosi (Romania); Hobi, et al. ^52^ and Trotsiuk, et al. ^53^ for *Fagus sylvatica* at Uholka (Ukraine); Commarmot, et al. ^54^ for *Fagus sylvatica* at Sihlwald (Switzerland); von Oheimb, et al. ^55^ for *Fagus sylvatica* at Serrahn (Germany); Wijdeven ^56^ for *Fagus sylvatica* at Fontainbleau (France); Merino, et al. ^57^ for *Fagus sylvatica* in northwestern Spain; Keeton, et al. ^58^ for *Abies alba* at Gorgany (Ukraine); Kreutz, et al. ^59^ for *Betula pendula* at Värriö (Finnland).

For each species, one representative study site was selected from the empirical study sites to run the simulations, which best represented old-growth forest conditions (i.e., as little anthropogenic disturbance as possible) as well as a clear predominance of the respective species of interest. Simulation results were compared with empirical data ranges for basal area and stem density, based on the approach characterizing old-growth forests by Heiri, et al. ^60^.

The location of the sites across Europe is shown in Fig.S1.1; further site-specific environmental conditions are provided in Table S1.2. The representation of site-specific environmental conditions for the ForClim simulations was either based on previous ForClim studies ^4,14^, or derived from published literature from the respective sites (see Table S1.2). Climate data for the study sites Perućica, Boky, Uholka and Värriö were derived from the Climate EU v 4.63 software package, available at http://tinyurl.com/ClimateEU, based on methodology described by Hamann, et al. ^61^. Due to the lack of field data about soil nitrogen availability at these sites, an averaged value of 80 kg ha^-1^ yr^-1^ was assumed based on Bircher ^62^.


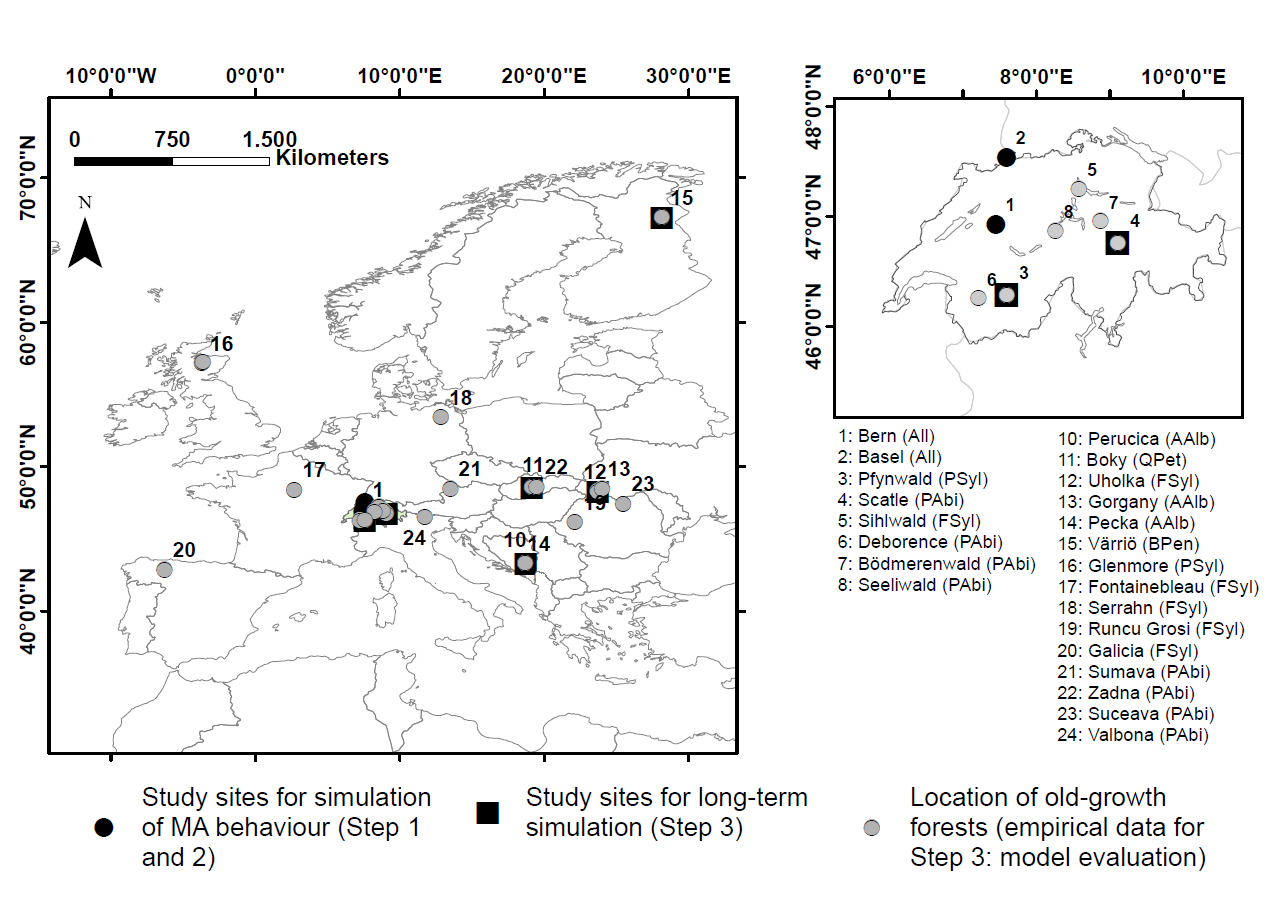


**Figure S1.1:** Location of simulation study sites and old-growth forests used for MA evaluation at the European scale (left panel) and Swiss scale (right panel). Point shading and shape indicate type if sute, point number refers to site name (see legend). All: Analysis of all species; PSyl: *Pinus sylvestris*; PAbi: *Picea abies*; AAlb: *Abies alba*; BPen: *Betula pendula*; QPet: *Quercus petraea*; FSyl: *Fagus sylvatica*. Sources of empirical data at respective sites were: 3: Brang and Heiri ^41^; 4,5,6,7,8: Rimle ^43^; 10, 14: Leibundgut ^48^; 5: Commarmot, et al. ^54^; 11: Korpel ^49^; 12: Hobi, et al. ^52^, Trotsiuk, et al. ^53^; 13: Keeton, et al. ^58^; 15: Kreutz, et al. ^59^; 16: Mason, et al. ^42^; 17: Wijdeven ^56^; 18: von Oheimb, et al. ^55^; 19: Petritan, et al. ^51^; 20: Merino, et al. ^57^; 21: Svoboda and Pouska ^44^; 22: Holeksa, et al. ^45^; 23: Lamedica, et al. ^46^; 24: Motta, et al. ^47^. ArcGIS Desktop (Version 10.8.1 (<https://www.esri.com/en-us/arcgis/products/arcgis-desktop/overview>) was used to create the map.

**Table S1.2** Site conditions (elevation, mean annual temperature, annual precipitation sum, soil water holding capacity (SWHC) and available nitrogen content (AvN)) at the study sites. Further site-specific information is given in the respective references.

| Site | Latitude | Longitude | Elevation (m a.s.l.) | Temp. (°C) | Prec. (mm) | Observation period | SWHC (cm) | AvN (kg ha^-1^ yr^-1^) | References |
| --- | --- | --- | --- | --- | --- | --- | --- | --- | --- |
|  |  |  |  |  |  |  |  |  |  |
| Bern | 46° 56' | 7° 26' | 570 | 8.4 | 1006 | 1864-2003 | 20 | 60 | Rasche, et al. ^14^ |
| Basel | 47° 33′ | 7° 35′ | 317 | 9.2 | 784 | 1880-2003 | 12 | 80 | Rasche, et al. ^14^ |
| Pfynwald (Sion) | 46° 18' | 7° 35' | 600 | 9.7 | 597 | 1864-2003 | 15 | 60 | Rasche, et al. ^14^ |
| Scatlè | 46°47' | 9°3' | 1510 | 3.41 | 1570 | 1930-2011 | 10 | 80 | Bircher, et al. ^4^ |
| Perućica | 43° 18' | 18° 42′ | 690 | 8.6 | 1428 | 1980-2009 | 15 | 80 | Leibundgut ^48^ |
| Boky | 48° 34′ | 19° 2′ | 300 | 7.5 | 720 | 1980-2009 | 12 | 80 | Korpel ^49^ |
| Uholka | 48° 16′ | 23° 40′ | 1500 | 7 | 950 | 1980-2009 | 20 | 80 | Commarmot, et al. ^54^ |
| Värriö | 67° 44′ | 29° 38′ | 100 | -0.9 | 570 | 1980-2009 | 12 | 80 | Kreutz, et al. ^59^ |
|  |  |  |  |  |  |  |  |  |  |

**Climate change scenarios**

For the simulations under changing climatic conditions, a set of simplified, hypothetical climate change scenarios was used ^63^, featuring clearly defined temperature and precipitation signals, which facilitates the analysis of MA responses to climate change. Following the approach of Bircher, et al. ^4^, temperature and precipitation conditions were assumed to change linearly over the course of 100 years (corresponding to the IPCC scenario timeframe, see IPCC ^64^) and remain at these new climatic conditions for the rest of the simulation period. Three climate change scenarios were assumed, a ‘warmer’ scenario (temperature increase by 4°C, precipitation remaining at present conditions), a ‘warmer and moister’ scenario (temperature increase by 4°C, precipitation increase by 20%) and a ‘warmer and drier’ scenario (temperature increase by 4°C, precipitation decrease by 20%). Standard deviations and cross-correlations between temperature and precipitation were assumed to stay constant at the level of present climate during climate change simulations ^4^. The assumption of a temperature increase by 4°C and a precipitation decrease by 20% was based on the ‘high impact’ RCP 8.5 projections until the end of the 21^st^ century for Central Europe ^64^. The assumption of a precipitation increase by 20% for the ‘warmer and moister’ scenario was based on the relatively large uncertainty regarding precipitation changes until the end of the 21^st^ century, which include the possibility of a substantial precipitation increase at this order of magnitude ^64^.

**Supplementary material S2 – Additional Results**

**Figure S2.1** Comparison of model behaviour based on the projected tree cohort half-life times (MT_50%_, time until 50% of the initially present trees died) and the response to competition (ΔMT_50%_, i.e., the relative percentage change in MT_50%_ for an increase in BAL by 10 m^2^ ha^-1^ relative to a ‘no competition’ scenario) for the six different tree species at the more xeric study site (Basel). Note that for response to competition, positive values indicate a relative increase in mortality and negative values a decrease in mortality. Symbol size indicates initial tree sizes used on the simulation, and symbol color indicates MA structure (differentiating between ‘Growth-based’ and ‘Competition-Index’ (CI)-based and ‘Size-only’ based MAs). Numbers refer the MA sources: 1: ForClim default MA (based on theoretical assumptions ^4^) , 2: Hülsmann, et al. ^38^, 3: Eid and Tuhus ^39^, 4: Monserud and Sterba ^32^, 5: Dursky ^31^, 6: Holzwarth, et al. ^26^, 7: Trasobares, et al. ^35^, 8: Crecente-Campo, et al. ^37^, 9: Palahi, et al. ^34^, 10:Bravo-Oviedo, et al. ^36^, 11: Fridman and Ståhl ^33^

**Figure S2.2** Mean change of MT_50%_ (ΔMT_50%_ in percentage) under future climate relative to present climate conditions (for BAL of 10 m^2^/ha and ‘Medium size’ of initial trees) for different tree species (in order of descending drought tolerance, based on Huber, et al. ^15^) at the site ‘Bern’. Note that for ΔMT_50%_, positive values indicate more mortality and negative values less mortality compared to the present climate conditions. Bars indicate ranges between min and max ΔMT_50%_ values of different MAs, no bars indicate that only one MA was present in the respective group. ‘No change’ indicates a change of ≤ 1%.

**Figure S2.3** Mean change of MT_50%_ (ΔMT_50%_ in percentage) for different tree species under a ‘warmer‘ climate change scenario (4°C temperature increase, precipitation remaining at present climatic conditions) for a range of competition (i.e. basal area of larger trees, BAL) and initial tree sizes (Small: 5 cm, Intermediate: 20 cm, Large: 40 cm of DBH) at the mesic study site (Bern). Point shapes indicate the MA type and colours indicate the direction of change (with positive values indicating an increase and negative values a decrease in mortality).

**Figure S2.4** Mean change of MT_50%_ (ΔMT_50%_ in percentage) for different tree species under a ‘warmer, moister‘ climate change scenario (4°C temperature increase and 20% precipitation increase) for a range of competition (i.e. basal area of larger trees, BAL) and initial tree sizes (Small: 5 cm, Intermediate: 20 cm, Large: 40 cm of DBH) at the mesic study site (Bern). Point shapes indicate the MA type and colours indicate the direction of change (with positive values indicating an increase and negative values a decrease in mortality).

**Figure S2.5** Mean change of MT_50%_ (ΔMT_50%_ in percentage) for different tree species under a ‘warmer, drier‘ climate change scenario (4°C temperature increase and 20% precipitation decrease) for a range of competition (i.e. basal area of larger trees, BAL) and initial tree sizes (Small: 5 cm, Intermediate: 20 cm, Large: 40 cm of DBH) at the mesic study site (Bern). Point shapes indicate the MA type and colours indicate the direction of change (with positive values indicating an increase and negative values a decrease in mortality).

**Figure S2.6** Mean change of MT_50%_ (ΔMT_50%_ in percentage) for different tree species under a ‘warmer‘ climate change scenario (4°C temperature increase and precipitation remaining at present climatic conditions) for a range of competition (i.e. basal area of larger trees, BAL) and initial tree sizes (Small: 5 cm, Intermediate: 20 cm, Large: 40 cm of DBH) at the more xeric study site (Basel). Point shapes indicate the MA type and colours indicate the direction of change (with positive values indicating an increase and negative values a decrease in mortality).

**Figure S2.7** Mean change of MT_50%_ (ΔMT_50%_ in percentage) for different tree species under a ‘warmer, moister‘ climate change scenario (4°C temperature increase and 20% precipitation increase) for a range of competition (i.e. basal area of larger trees, BAL) and initial tree sizes (Small: 5 cm, Intermediate: 20 cm, Large: 40 cm of DBH) at the more xeric study site (Basel). Point shapes indicate the MA type and colours indicate the direction of change (with positive values indicating an increase and negative values a decrease in mortality).

**Figure S2.8** Mean change of MT_50%_ (ΔMT_50%_ in percentage) for different tree species under a ‘warmer, drier‘ climate change scenario (4°C temperature increase and 20% precipitation decrease) for a range of competition (i.e. basal area of larger trees, BAL) and initial tree sizes (Small: 5 cm, Intermediate: 20 cm, Large: 40 cm of DBH) at the more xeric study site (Basel). Point shapes indicate the MA type and colours indicate the direction of change (with positive values indicating an increase and negative values a decrease in mortality).

| Species | MA-Type | n | Basal area | | Stem density | |
| --- | --- | --- | --- | --- | --- | --- |
|  |  |  | PB [%] | (sd) | PB [%] | (sd) |
| *Pinus sylvestris* | Growth-based | 3 | **4** | 30 | **10** | 73 |
|  | CI-based | 5 | 147 | 113 | -62 | 29 |
|  |  |  |  |  |  |  |
| *Picea abies* | Growth-based | 2 | **-12** | 39 | 50 | 129 |
|  | CI-based | 3 | 28 | 81 | **-45** | 13 |
|  |  |  |  |  |  |  |
| *Abies alba* | Growth-based | 2 | 79 | 67 | **51** | 26 |
|  | Size-only-based | 1 | **34** | - | -61 | - |
|  |  |  |  |  |  |  |
| *Betula pendula* | Growth-based | 2 | **18** | 61 | **-15** | 13 |
|  | CI-based | 1 | 509 | - | 159 | - |
|  | Size-only-based | 1 | 55 | - | -48 | - |
|  |  |  |  |  |  |  |
| *Quercus petraea* | Growth-based | 2 | **-1** | 33 | 184 | 219 |
|  | Size-only-based | 1 | 21 | - | **71** | - |
|  |  |  |  |  |  |  |
| *Fagus sylvatica* | Growth-based | 4 | **103** | 72 | **23** | 47 |
|  | CI-based | 1 | 159 | - | -70 | - |
|  |  |  |  |  |  |  |
| All Species | Growth-based | 15 | **36** | 65 | 44 | 100 |
|  | CI-based | 10 | 162 | 163 | -35 | 76 |
|  | Size-only-based | 3 | 37 | 17 | **-13** | 73 |
|  |  |  |  |  |  |  |

**Table. S2.9** Model-Data comparison between ‘dynamic equilibrium’ simulations and measured data from old-growth forests. Percentage bias (PB) is provided as a measure for deviation between model and data, with positive numbers indicating an overprediction and negative numbers an underprediction of basal area or stem density by the respective MA. The reference for calculating the PB was the mean of basal area and of stem number measured at the old-growth forests of the respective species.

**References**

1 Bugmann, H. A review of forest gap models. *Climatic Change* **51**, 259-305, doi:10.1023/A:1012525626267 (2001).

2 Shugart, H. H. & Smith, T. M. A review of forest patch models and their application to global change research. *Climatic Change* **34**, 131-153, doi:10.1007/BF00224626 (1996).

3 Watt, A. S. Pattern and Process in the Plant Community. *Journal of Ecology* **35**, 1-22, doi:10.2307/2256497 (1947).

4 Bircher, N., Cailleret, M. & Bugmann, H. The agony of choice: different empirical mortality models lead to sharply different future forest dynamics. *Ecological Applications* **25**, 1303-1318, doi:10.1890/14-1462.1 (2015).

5 Bugmann, H. A simplified forest model to study species composition along climate gradients. *Ecology* **77**, 2055-2074, doi:10.2307/2265700 (1996).

6 Monsi, M. & Saeki, T. On the Factor Light in Plant Communities and its Importance for Matter Production. *Annals of Botany* **95**, 549-567, doi:10.1093/aob/mci052 (2005).

7 Bugmann, H. & Cramer, W. Improving the behaviour of forest gap models along drought gradients. *Forest Ecology and Management* **103**, 247-263, doi:10.1016/S0378-1127(97)00217-X (1998).

8 Rasche, L. *Bridging the gap between forest growth and forest succession models. PhD Thesis* No 20173 , ETH Zurich (2012).

9 Friend, A. D., Stevens, A. K., Knox, R. G. & Cannell, M. G. R. A process-based, terrestrial biosphere model of ecosystem dynamics (Hybrid v3.0). *Ecological Modelling* **95**, 249-287, doi:10.1016/S0304-3800(96)00034-8 (1997).

10 Sitch, S. *et al.* Evaluation of ecosystem dynamics, plant geography and terrestrial carbon cycling in the LPJ dynamic global vegetation model. *Global Change Biology* **9**, 161-185, doi:DOI 10.1046/j.1365-2486.2003.00569.x (2003).

11 Sato, H., Itoh, A. & Kohyama, T. SEIB-DGVM: A new dynamic global vegetation model using a spatially explicit individual-based approach. *Ecological Modelling* **200**, 279-307, doi:10.1016/j.ecolmodel.2006.09.006 (2007).

12 Bugmann, H. *On the ecology of mountainous forests in a changing climate: a simulation study. Ph.D. Thesis no. 10638*, ETH Zurich (1994).

13 Huber, N. *Towards robust projections of future forest dynamics: why there is no silver bullet to cope with complexity. PhD Thesis* no 26061, ETH Zurich (2019).

14 Rasche, L., Fahse, L., Zingg, A. & Bugmann, H. Enhancing gap model accuracy by modeling dynamic height growth and dynamic maximum tree height. *Ecological Modelling* **232**, 133-143, doi:10.1016/j.ecolmodel.2012.03.004 (2012).

15 Huber, N., Bugmann, H. & Lafond, V. Capturing ecological processes in dynamic forest models: why there is no silver bullet to cope with complexity. *Ecosphere* **11**, 1-34, doi:10.1002/ecs2.3109 (2020).

16 Shao, G. F., Bugmann, H. & Yan, X. D. A comparative analysis of the structure and behavior of three gap models at sites in northeastern China. *Climatic Change* **51**, 389-413, doi:Doi 10.1023/A:1012550300768 (2001).

17 Gutierrez, A. G., Snell, R. S. & Bugmann, H. Using a dynamic forest model to predict tree species distributions. *Global Ecology and Biogeography* **25**, 347-358, doi:10.1111/geb.12421 (2016).

18 Huber, N., Bugmann, H. & Lafond, V. Global sensitivity analysis of a dynamic vegetation model: Model sensitivity depends on successional time, climate and competitive interactions. *Ecological Modelling* **368**, 377-390, doi:10.1016/j.ecolmodel.2017.12.013 (2018).

19 Grimm, V. Pattern-Oriented Modeling of Agent-Based Complex Systems: Lessons from Ecology. *Science* **310**, 987-991, doi:10.1126/science.1116681 (2005).

20 Didion, M., Kupferschmid, A. D. & Bugmann, H. Long-term effects of ungulate browsing on forest composition and structure. *Forest Ecology and Management* **258**, S44-S55, doi:10.1016/j.foreco.2009.06.006 (2009).

21 Mina, M., Bugmann, H., Klopcic, M. & Cailleret, M. Accurate modeling of harvesting is key for projecting future forest dynamics: a case study in the Slovenian mountains. *Regional Environmental Change* **17**, 49-64, doi:10.1007/s10113-015-0902-2 (2017).

22 Thrippleton, T., Hülsmann, L., Cailleret, M. & Bugmann, H. Projecting forest dynamics across Europe: potentials and pitfalls of empirical mortality algorithms. *Ecosystems* **23**, 188-203, doi:10.1007/s10021-019-00397-3 (2020).

23 Moore, A. D. On the Maximum Growth Equation Used in Forest Gap Simulation-Models. *Ecological Modelling* **45**, 63-67, doi:10.1016/0304-3800(89)90100-2 (1989).

24 Manusch, C., Bugmann, H., Heiri, C. & Wolf, A. Tree mortality in dynamic vegetation models - A key feature for accurately simulating forest properties. *Ecological Modelling* **243**, 101-111, doi:DOI 10.1016/j.ecolmodel.2012.06.008 (2012).

25 Lines, E. R., Coomes, D. A. & Purves, D. W. Influences of Forest Structure, Climate and Species Composition on Tree Mortality across the Eastern US. *Plos One* **5**, 1-12, doi:10.1371/journal.pone.0013212 (2010).

26 Holzwarth, F., Kahl, A., Bauhus, J. & Wirth, C. Many ways to die - partitioning tree mortality dynamics in a near-natural mixed deciduous forest. *Journal of Ecology* **101**, 220-230, doi:10.1111/1365-2745.12015 (2013).

27 Solomon, A. M. Transient-Response of Forests to Co2-Induced Climate Change - Simulation Modeling Experiments in Eastern North-America. *Oecologia* **68**, 567-579, doi: 10.1007/Bf00378773 (1986).

28 Monserud, R. A. Simulation of Forest Tree Mortality. *Forest Science* **22**, 438-444, doi:10.1093/forestscience/22.4.438 (1976).

29 Weiskittel, A. R., Hann, D. W., Kershaw, J. A. & Vanclay, J. K. in *Forest Growth and Yield Modeling* Ch. 8, 139-155 (John Wiley & Sons, 2011).

30 Hülsmann, L., Bugmann, H. & Brang, P. How to predict tree death from inventory data - lessons from a systematic assessment of European tree mortality models. *Canadian Journal of Forest Research* **47**, 890-900, doi:10.1139/cjfr-2016-0224 (2017).

31 Dursky, J. Modellierung der Absterbeprozesse in Rein- und Mischbeständen aus Fichte und Buche. *Allg. Forst- u. Jagdztg.* **168**, 131-134 (1997).

32 Monserud, R. A. & Sterba, H. Modeling individual tree mortality for Austrian forest species. *Forest Ecology and Management* **113**, 109-123, doi:10.1016/S0378-1127(98)00419-8 (1999).

33 Fridman, J. & Ståhl, G. A three-step approach for modelling tree mortality in Swedish forests. *Scandinavian Journal of Forest Research* **16**, 455-466, doi:10.1080/02827580152632856 (2001).

34 Palahi, M., Pukkala, T., Miina, J. & Montero, G. Individual-tree growth and mortality models for Scots pine (Pinus sylvestris L.) in north-east Spain. *Annals of Forest Science* **60**, 1-10, doi:10.1051/forest:2002068 (2003).

35 Trasobares, A., Pukkala, T. & Muna, J. Growth and yield model for uneven-aged mixtures of Pinus sylvestris L. and Pinus nigra Arn. in Catalonia, north-east Spain. *Annals of Forest Science* **61**, 9-24, doi:10.1051/forset:2003080 (2004).

36 Bravo-Oviedo, A., Sterba, H., del Rio, M. & Bravo, F. Competition-induced mortality for Mediterranean Pinus pinaster Ait. and P-sylvestris L. *Forest Ecology and Management* **222**, 88-98, doi:10.1016/j.foreco.2005.10.016 (2006).

37 Crecente-Campo, F., Soares, P., Tome, M. & Dieguez-Aranda, U. Modelling annual individual-tree growth and mortality of Scots pine with data obtained at irregular measurement intervals and containing missing observations. *Forest Ecology and Management* **260**, 1965-1974, doi:10.1016/j.foreco.2010.08.044 (2010).

38 Hülsmann, L., Bugmann, H., Cailleret, M. & Brang, P. How to kill a tree: empirical mortality models for 18 species and their performance in a dynamic forest model. *Ecological Applications* **28**, 522-540, doi:10.1002/eap.1668 (2018).

39 Eid, T. & Tuhus, E. Models for individual tree mortality in Norway. *Forest Ecology and Management* **154**, 69-84, doi:10.1016/S0378-1127(00)00634-4 (2001).

40 Bugmann, H. *et al.* in *Towards Quantitative Scenarios of Climate Change Impacts in Switzerland* (ed CH2014-Impacts) (OCCR, FOEN, MeteoSwiss, C2SM, Agroscope, and ProClim, 2014).

41 Brang, P. & Heiri, C. in *Waldreservate - 50 Jahre natürliche Waldentwicklung in der Schweiz* (eds P.; Brang, C.; Heiri, & H. Bugmann) Ch. 6.5, 140-149 (Paul Haupt Verlag, 2011).

42 Mason, W. L., Connolly, T., Pommerening, A. & Edwards, C. Spatial structure of semi-natural and plantation stands of Scots pine (Pinus sylvestris L.) in northern Scotland. *Forestry* **80**, 564-583, doi:10.1093/forestry/cpm038 (2007).

43 Rimle, A. *Quantity and quality of coarse woody debris in mountainous Norway spruce forest reserves in Switzerland*, Msc-Thesis ETH Zurich, doi:10.3929/ethz-a-010685934 (2016).

44 Svoboda, M. & Pouska, V. Structure of a Central-European mountain spruce old-growth forest with respect to historical development. *Forest Ecology and Management* **255**, 2177-2188, doi:10.1016/j.foreco.2007.12.031 (2008).

45 Holeksa, J. *et al.* Altitudinal variability of stand structure and regeneration in the subalpine spruce forests of the Pol'ana biosphere reserve, Central Slovakia. *Eur. J. For. Res.* **126**, 303-313, doi:10.1007/s10342-006-0149-z (2007).

46 Lamedica, S., Lingua, E., Popa, I., Motta, R. & Carrer, M. Spatial Structure in Four Norway Spruce Stands with Different Management History in the Alps and Carpathians. *Silva. Fenn.* **45**, 865-873, doi:10.14214/sf.75 (2011).

47 Motta, R. *et al.* Stand and coarse woody debris dynamics in subalpine Norway spruce forests withdrawn from regular management. *Annals of Forest Science* **67**, doi:10.1051/forest/2010040 (2010).

48 Leibundgut, H. *Europäische Urwälder - Wegweiser zur naturnahen Waldwirtschaft*. (Paul Haupt Verlag, 1993).

49 Korpel, Š. *Die Urwälder der Westkarpaten*. 310 (Gustav Fischer, 1995).

50 Bobiec, A. Bialowieza Primeval Forest as a remnant of culturally modified ancient forest. *Eur. J. For. Res.* **131**, 1269-1285, doi:10.1007/s10342-012-0597-6 (2012).

51 Petritan, A. M., Biris, I. A., Merce, O., Turcu, D. O. & Petritan, I. C. Structure and diversity of a natural temperate sessile oak (Quercus petraea L.) - European Beech (Fagus sylvatica L.) forest. *Forest Ecology and Management* **280**, 140-149, doi:10.1016/j.foreco.2012.06.007 (2012).

52 Hobi, M. L., Commarmot, B. & Bugmann, H. Pattern and process in the largest primeval beech forest of Europe (Ukrainian Carpathians). *Journal of Vegetation Science* **26**, 323-336, doi:10.1111/Jvs.12234 (2015).

53 Trotsiuk, V., Hobi, M. L. & Commarmot, B. Age structure and disturbance dynamics of the relic virgin beech forest Uholka (Ukrainian Carpathians). *Forest Ecology and Management* **265**, 181-190, doi:10.1016/j.foreco.2011.10.042 (2012).

54 Commarmot, B. *et al.* Structures of virgin and managed beech forests in Uholka (Ukraine) and Sihlwald (Switzerland): a comparative study. *For. Snow Landsc. Res.* **79**, 45-56 (2005).

55 von Oheimb, G., Westphal, C., Tempel, H. & Härdtle, W. Structural pattern of a near-natural beech forest (Fagus sylvatica) (Serrahn, North-east Germany). *Forest Ecology and Management* **212**, 253-263, doi:10.1016/j.foreco.2005.03.033 (2005).

56 Wijdeven, S. M. J. Stand dynamics in Fontainebleau. Dynamics in beech forest structure and composition over 17 years in La Tillaie forest reserve, Fontainebleau, France. 56 (Alterra, Green World Research, Wageningen, 2003).

57 Merino, A., Real, C., Alvarez-Gonzalez, J. G. & Rodriguez-Guitian, M. A. Forest structure and C stocks in natural Fagus sylvatica forest in southern Europe: The effects of past management. *Forest Ecology and Management* **250**, 206-214, doi:10.1016/j.foreco.2007.05.016 (2007).

58 Keeton, W. S. *et al.* Structural characteristics and aboveground biomass of old-growth spruce-fir stands in the eastern Carpathian mountains, Ukraine. *Plant Biosyst* **144**, 148-159, doi:10.1080/11263500903560512 (2010).

59 Kreutz, A., Aakala, T., Grenfell, R. & Kuuluvainen, T. Spatial tree community structure in three stands across a forest succession gradient in northern boreal Fennoscandia. *Silva. Fenn.* **49** doi: 10.14214/sf.1279 (2015).

60 Heiri, C., Brang, P., Commarmot, B., Matter, J.-F. & Bugmann, H. in *Waldreservate. 50 Jahre natürliche Waldentwicklung in der Schweiz* (eds P.; Brang, C.; Heiri, & H. Bugmann) 72-89 (Eidg. Forschungsanstalt WSL; Zürich, ETH Zürich; Haupt., 2011).

61 Hamann, A., Wang, T. L., Spittlehouse, D. L. & Murdock, T. Q. A Comprehensive, High-Resolution Database of Historical and Projected Climate Surfaces for Western North America. *B Am Meteorol Soc* **94**, 1307-1309, doi:10.1175/Bams-D-12-00145.1 (2013).

62 Bircher, N. *To die or not to die - Forest dynamics in Switzerland under climate change. PhD thesis No 22775*, ETH Zurich, (2015).

63 Bugmann, H. K. M. *et al.* Comparing the performance of forest gap models in North America. *Climatic Change* **51**, 349-388, doi:10.1023/A:1012537914881 (2001).

64 IPCC. Climate Change 2013: The Physical Science Basis. Contribution of Working Group I to the Fifth Assessment Report of the Intergovernmental Panel on Climate Change. (Cambridge University Press, Cambridge, United Kingdom and New York, NY, USA, 2013).
